# Application of Two Blastocyst Biopsy Strategies in Preimplantation Genetic Testing Treatment and Assessment of Their Effects

**DOI:** 10.3389/fendo.2022.852620

**Published:** 2022-03-04

**Authors:** Han Yang, Dandan Yang, Qi Zhu, Kaijuan Wang, Chao Zhang, Beili Chen, Weiwei Zou, Yan Hao, Ding Ding, Zhaojuan Yu, Dongmei Ji, Dawei Chen, Yunxia Cao, Huijuan Zou, Zhiguo Zhang

**Affiliations:** ^1^ Department of Biomedical Engineering, Anhui Medical University, Hefei, China; ^2^ Reproductive Medicine Center, Department of Obstetrics and Gynecology, The First Affiliated Hospital of Anhui Medical University, Hefei, China; ^3^ National Health Commission (NHC) Key Laboratory of Study on Abnormal Gametes and Reproductive Tract (Anhui Medical University), Hefei, China; ^4^ Biopreservation and Artificial Organs, Anhui Provincial Engineering Research Center, Anhui Medical University, Hefei, China

**Keywords:** blastocyst biopsy, clinical outcomes, embryo development, human-assisted reproductive technology, next-generation sequencing, preimplantation genetic testing

## Abstract

**Background:**

Blastocyst biopsy has become the most mainstream biopsy method. Currently, there are two blastocyst biopsy strategies. Many studies have compared the advantages and disadvantages between blastomere and blastocyst biopsy, but fewer articles have compared the two blastocyst biopsy strategies. For the moment, no published studies have explored the entire set of information on embryo development, next-generation sequencing results, and clinical outcomes, including the baby’s health status with the two blastocyst biopsy strategies.

**Methods:**

A total of 323 preimplantation genetic testing cycles from April 2018 to May 2020, including 178 cycles with Strategy A and 145 cycles with Strategy B. Strategy A was to create a laser-assisted zona pellucid opening for cleavage embryo on the third day after insemination, but Strategy B was not. Strategy A performed a biopsy for artificially assisted hatching blastocysts, while Strategy B performed a biopsy for expanded blastocysts on day 5 or 6. In this study, embryo development, next-generation sequencing results, pregnancy outcomes, and offspring health of the two strategies were compared and analyzed.

**Results:**

There were no statistical differences between the two groups in the rate of fertilization, blastocyst and abortion. The rate of cleavage from Strategy A was slightly higher than Strategy B, and the rate of high-quality cleavage embryo was lower than Strategy B, while the rate of high-quality blastocyst was higher than Strategy B. The rate of no-results blastocyst was significantly lower than Strategy B. In particular, the rate of biochemical pregnancy, clinical pregnancy, and live birth of Strategy A were significantly lower than those of Strategy B. The average Apgar scores of newborns were ≥8 in both groups, and there was no significant difference in average height and weight. In Strategy A, a baby was born with thumb syndactyly, and Strategy B had no congenital disabilities.

**Conclusions:**

Blastocyst biopsy strategy without laser-assisted zona pellucid drilling on day 3 achieves better clinical treatment effects. Therefore, Strategy B is an optimal treatment regime for PGT.

## 1 Introduction

Preimplantation genetic testing (PGT) is one of the essential techniques in human-assisted reproductive technology (ART), which contributes to reducing the transmission of genetic diseases. With the growth of women’s age, especially after 40 years, the probability of embryo aneuploidy increases dramatically, which easily leads to implantation failure or miscarriage (1, 2). PGT is to identify embryos with normal chromosomes for transfer, containing Preimplantation genetic testing for aneuploidy (PGT-A), Preimplantation genetic testing for monogenic/single gene defects (PGT-M), and Preimplantation genetic testing for structural rearrangements (PGT-SR). It can significantly increase the success rate of ART. The first PGT baby was born in 1990, in which to avoid the transmission of recessive X chromosome disease to male offspring, DNA amplification was used to screen out female embryos for transfer, and finally, healthy female twins were delivered successfully (3).

Currently, the biopsy methods used in the clinic mainly include cleavage embryo biopsy, blastocyst biopsy, and polar body biopsy. Cleavage embryo biopsy extracts 1-2 blastomeres from the embryo containing 6 cells or more on the third day after insemination. Blastocyst biopsy is a dissection method of trophectoderm (TE) cells from blastocysts, usually performed on day 5 or 6. Polar body biopsy is to analyze the first polar body of mature oocytes or the second polar body of fertilized eggs. It is a diagnostic method for maternally derived genetic defects, but it cannot assess paternal factors (4). Recent studies have extracted DNA and blastocoel fluid from the conditioned blastocyst culture medium to verify the euploidy of chromosomes. In this way, non-invasive preimplantation genetic screening can be realized (5, 6). However, more research data are still insufficient for its application in clinical practice.

Many embryologists have studied the effect of biopsy methods on embryo safety and clinical outcomes. Kalma et al. demonstrated that blastomere biopsy performed 15-20 hours after the embryo develops to 8 cells is less harmful to the embryo (7). Chen Linjun et al. have shown that the blastocyst biopsy on day 5 after insemination has a higher embryo implantation rate and live birth rate than the day 6 (8). There are also many studies comparing cleavage stage biopsy and blastocyst stage biopsy, proving that blastomere biopsy significantly reduces the probability of embryo implantation and live birth, while blastocyst biopsy is relatively safer and has better clinical outcomes (9, 10). A growing number of reproductive medicine centers are using blastocyst biopsy. There are two blastocyst biopsy strategies currently. However, which method is more effective among the two blastocyst biopsy methods? This study compared the embryo development, NGS results, and clinical pregnancy outcomes of the two blastocyst stage biopsy strategies, hoping to provide a reference for this question.

In this research, the blastocyst biopsy method was applied to all PGT treatments. The cleavage embryos underwent laser hatching on the third day after insemination and left a hole, and then the laser was used again to biopsy TE cells herniating through that hole on day 5 or 6 (11, 12), which was called Strategy A here. The blastocysts reaching a morphologic grade of 4 with AA, AB, BA, or BB (also called as an expanded blastocyst) (13) were biopsied for TE cells on day 5 or 6 called Strategy B (14). The main difference between the two biopsy strategies is that Strategy A is to create a laser-assisted zona pellucida (ZP) opening for the cleavage embryo on the third day after insemination, while Strategy B does not. All biopsy samples in this study were assessed using next-generation sequencing (NGS). We analyzed the embryo development, NGS results, and clinical outcomes of the two strategies to determine which is safer and more effective.

## 2 Materials and Methods

### 2.1 Study Population

We practiced a total of 323 preimplantation genetic testing-thawed embryo transfer (PGT-TET) cycles at Reproductive Medicine Center of the First Affiliated Hospital of Anhui Medical University, from April 2018 to May 2020. Each patient was randomly assigned into one of the two strategies by lottery. There were 178 cycles of Strategy A and 145 cycles of Strategy B included, with 1187 embryos undergoing biopsy in Strategy A and 902 embryos in Strategy B (**Table 3**). Most patients were diagnosed with chromosomal abnormalities in one or both partners; recurrent miscarriage; abnormal gestation and birth or teratozoospermia.

### 2.2 Ethics Statement

The biological sample study was approved by the Medical Ethics Committee of Anhui Medical University (Ethics approval number: 2017002). All patients in this study had signed informed consent before PGT therapy cycles.

### 2.3 Oocyte Retrieval and ICSI

The female patients received classic controlled ovarian hyperstimulation program to promote ovulation. The specific drugs and procedures have been reported (15). Through the transvaginal ultrasound-guided oocyte retrieval, the cumulus-oocyte complexes were picked up from the follicular fluid under an inverted microscope. After ovum pick-up, the cumulus and corona cells were removed through the action of hyaluronidase solution (VitroLife, Gotebor, Sweden). Then, the mature oocytes were selected for intracytoplasmic sperm injection (ICSI). Operators with many years of experience performed all ICSI processes. The ICSI oocytes were cultured for three days in the environment containing 5% O_2_ and 6% CO_2_ at 37°C in a microdroplet (20-30 μL) of cleavage culture medium (COOK, Sydney, Australia), covered with mineral oil (VitroLife, Gotebor, Sweden). During the period, fertilization was evaluated 16 - 18 hours after ICSI. In this study, high-quality embryos refer to those reach 7–9 cells by day 3, with <15% fragmentation and no multinucleation, and have cleaved during the preceding 24 h (16). High-quality blastocysts refer to embryos which were ≥3BB on day 5 or ≥4BB on day 6 (13).

### 2.4 The Operation of Two Biopsy Strategies

#### 2.4.1 Strategy A

**Figure 1:**
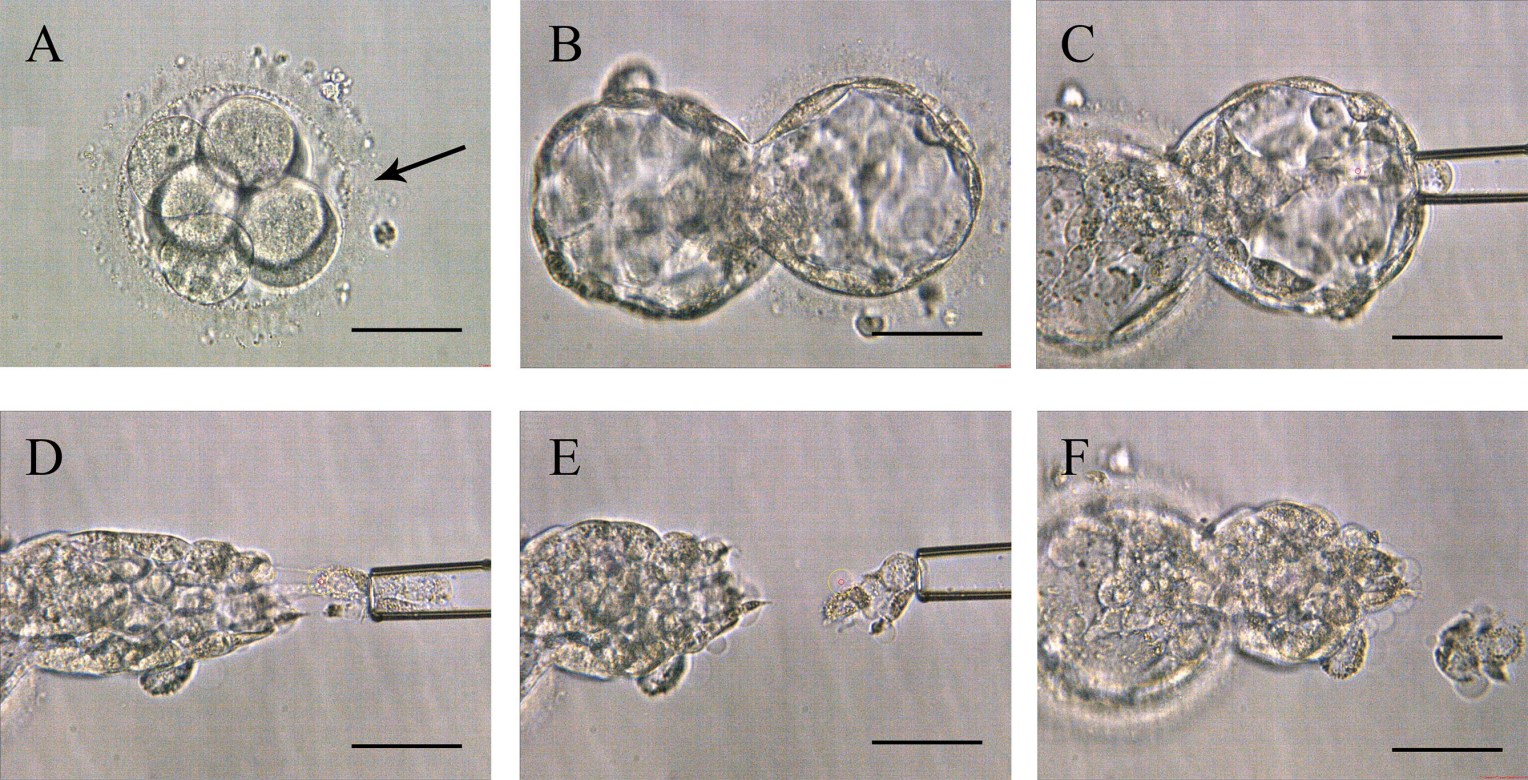
Procedures of Strategy A. **(A)** The zona pellucida was opened a 10-15 µm hole (pointed by the black arrow in the picture) by lasers to assist hatching on day 3. **(B)** Expanding blastocyst with trophectoderm cells herniating from the artificial opening on day 5 or 6. **(C)** The biopsy pipette sucked the herniating cells. **(D)** Disconnected the junction in front of the biopsy pipette with lasers. **(E)** Trophectoderm cell samples isolated from the embryo’s body. **(F)** Blastocyst morphology after biopsy. Scale bar = 50 µm.

Around 10:00 am on the third day, a ~10 µm hole in the ZP was made with a series of 500-μs laser pulses (Hamilton Thorne LYKOS, Beverly, MA, USA). Then the embryos were placed into a microdroplet of blastocyst medium (COOK, Sydney, Australia) covered with mineral oil and cultured to day 5 or 6 until the blastocyst was hatching. Embryo biopsy was performed around 11-12 am in a petri dish (Life Sciences, Durham, USA) containing 7.5 μL blastocyst medium. The holding pipette (Sunlight Medical Inc, Jacksonville, USA) was used to fix the hatching blastocyst at the 9 o’clock position. Then, 8-10 herniating TE cells were aspirated through the biopsy pipette (Sunlight Medical Inc, Jacksonville, USA) at the 3 o’clock position. The TE cells were disconnected with lasers along the flat mouth of biopsy pipette (**Figure 1** and **Video 1**). The cells to be detected were transferred into a 200 μL PCR tube containing 2 μL phosphate buffered saline (ThermoFisher Biochemical Products Co., Ltd, Beijing, China). All of the operations were performed on the heated micromanipulator (Nikon, eclipse Ti2, Japan). The post-biopsy blastocysts were cryopreserved by vitrification in liquid nitrogen for future TET.

#### 2.4.2 Strategy B

**Figure 2:**
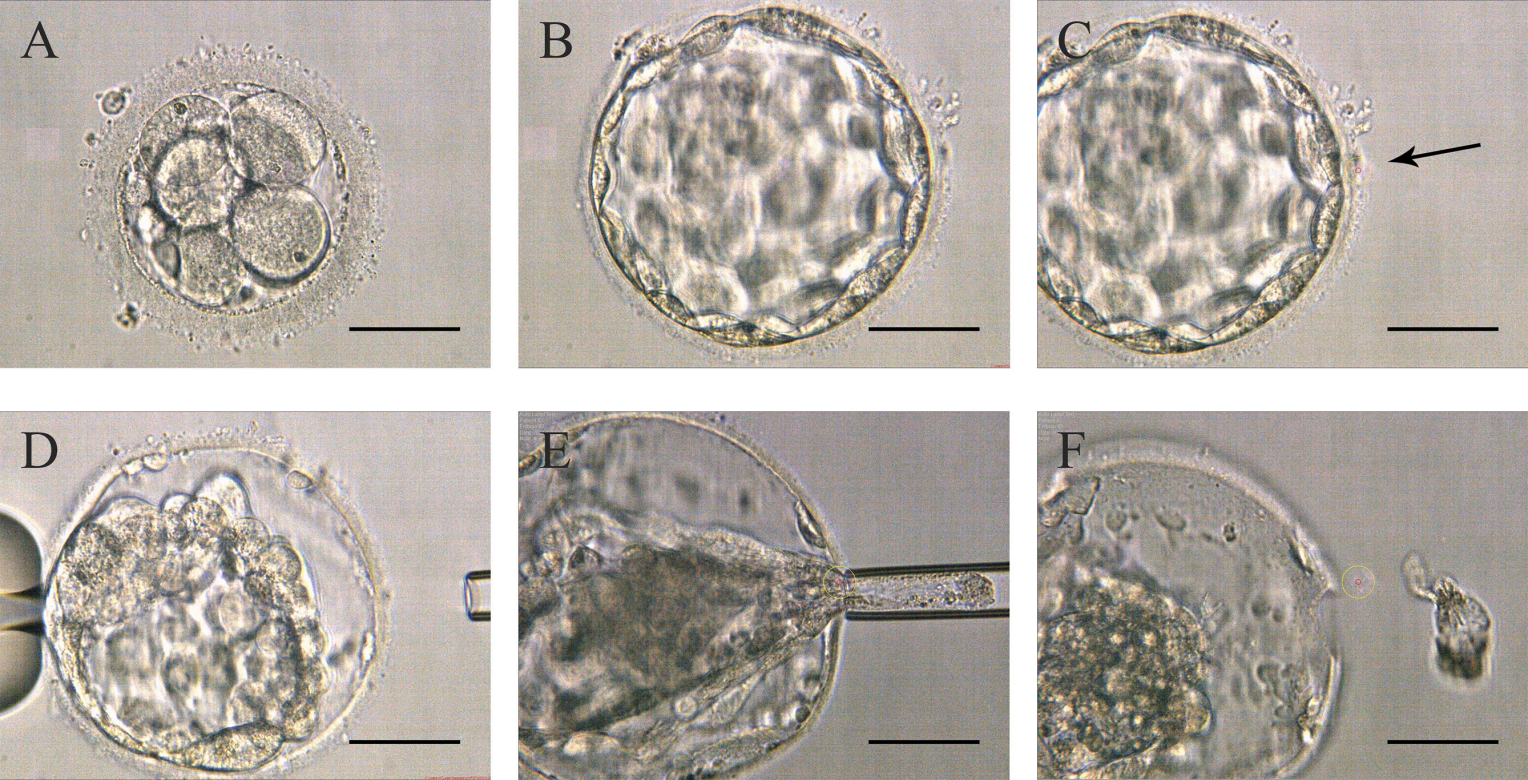
Procedures of Strategy B **(A)** Embryos on day 3 without zona pellucida opening. **(B)** High-quality blastocyst with an expansion grade of 4 with AA, AB, BA, or BB on day 5 or 6. **(C)** The zona pellucida was opened a small hole (pointed by the black arrow in the picture) to flow out blastocyst fluid. **(D)** After the trophectoderm cells shrunk, the lasers were used to make a hole in the zona pellucida. **(E)** The biopsy pipette entered the inner blastocyst to suck the cells out of the zona pellucida, and disconnected the junction in front of the biopsy pipette with lasers. **(F)** Trophectoderm cell samples isolated from the embryo’s body. Scale bar = 50 µm.

Around 10:00 am on the third day, the embryos were directly transferred to the microdroplet blastocyst medium covered with mineral oil and continued to be cultured until they reached the blastocyst stage with a morphologic grade of 4 (4AA, 4AB, 4BA, or 4BB) on day 5 or 6. The biopsy was performed around 11-12 am. Firstly, the expanded blastocyst was fixed with a holding pipette at the 9 o’clock position and then a small hole was left on the ZP with the assist of a laser. Secondly, the biopsy pipette was used to continuously press the blastocyst’s periphery until the TE shrunk and separated from the inner surface of the ZP. Subsequently, the laser was used again to drill an about 5 µm hole by multiple pulses, and the hole was far away from the inner cell mass (ICM). Finally, the biopsy pipette was inserted into the blastocyst through the hole, and the contracted TE was sucked tightly through the negative pressure, and then some of the TE cells are pulled out of the hole at the same time, the laser is emitted to cut TE cells along the flat mouth of the pipette (**Figure 2** and **Video 2**). The following steps were as described in Strategy A.

### 2.5 Embryo Selection and Transfer

The NGS was performed with an Ion Proton Sequencing (Life Technologies, Grand Island, NY, USA). High-quality blastocysts with normal chromosomes tested by PGT would be firstly recommended to thaw and transfer. If there was no euploid embryo with patients, mosaic embryos with mosaicism <30% could be transferred in our center. Single embryo transfer was used for all statistical cycles. A positive hCG value (≥25 IU/L) on day 14 after transplantation is a sign of biochemical pregnancy. The appearance of a pregnancy sac by ultrasound scanning is regarded as clinical pregnancy.

### 2.6 Statistical Analysis

Continuous variables were expressed as the mean ± SD (standard deviation), and categorical variables were evaluated by the Chi-square test (χ^2^). We used the χ^2^ test for the data of embryo development, NGS results, and clinical outcomes. The two-sample t-test for age, hormone values and neonatal health, including Apgar score, height, and weight. GraphPad Prism 8.0 software (GraphPad Software, San Diego, USA) was used for statistical analysis. *P*-values < 0.05 were considered statistically significant.

## 3 Results

In these cycles, the average age of the women in the two groups was about 30 years old, and the men was about 32 years old. In addition, the majority of the basal characteristics for all patients in the two groups are listed in **Table 1**. There were no significant differences in the baseline data between the two groups of patients.

**Table 1:** Baseline level of patients from the two biopsy strategies.

	Strategy A	Strategy B	t value	*P*-value
Female age (years)	30.59 ± 4.45	30.73 ± 4.54	0.280	0.779
Advanced female (≥35 years old) (%)	19.66% (35/178)	22.07% (32/145)	–	0.596
Male age (years)	32.39 ± 4.66	32.48 ± 5.81	0.152	0.880
BMI (kg/m^2^)	22.27 ± 2.81	22.06 ± 4.25	0.543	0.588
Basal FSH (IU/L)	6.78 ± 1.74	6.94 ± 2.06	0.783	0.434
Basal E_2_ (IU/L)	151.40 ± 92.47	155.80 ± 138.80	0.339	0.735
Basal P (IU/L)	3.00 ± 6.51	3.09 ± 8.16	0.110	0.912
Basal PRL (IU/L)	24.29 ± 46.66	35.90 ± 86.11	1.543	0.124
Basal LH (IU/L)	5.28 ± 3.32	6.28 ± 9.24	1.348	0.179
Basal T (IU/L)	1.95 ± 4.78	2.98 ± 7.80	1.453	0.147
PGT-A (%)	41.57% (74/178)	46.21% (67/145)	–	0.404
PGT-SR (%)	48.31% (86/178)	46.90% (68/145)	–	0.800
PGT-M (%)	10.11% (18/178)	6.90% (10/145)	–	0.307

BMI, body mass index; FSH, follicle-stimulating hormone; E_2_, estrogenic hormone; P, progestational hormone; PRL, prolactin; LH, luteinizing hormone; T, testosterone.

We analyzed the embryo development, NGS results, and clinical outcomes of all PGT-TET with complete information from April 2018 to May 2020. The fertilization rate of Strategy A was slightly lower than that of Strategy B, with no statistical difference (82.74% vs 83.37%; *P*=0.547). Strategy A had a higher cleavage rate (98.73%) than Strategy B (97.69%). Although there was a statistical difference (*P*=0.01), both were above 97%, which may be related to the quality of patients’ oocytes and other factors. There were no significant differences in the blastocyst rate of the two strategies, both are above 50% (53.51% vs 52.56%; *P*=0.546). However, the high-quality blastocyst rate was obviously higher in Strategy A groups (47.73% vs 42.31%; *P*<0.001) (**Table 2**).

**Table 2:** Embryo development and clinical outcomes.

	Strategy A	Strategy B	*P*-value
Fertilization (%)	82.74% (2364/2857)	83.37% (1817/2179)	0.547
Cleavage (%)	98.73% (2334/2364)	97.69% (1775/1817)	0.010
High-quality embryo on day 3 (%)	48.46% (1131/2334)	52.06% (924/1775)	0.022
Blastocyst (%)	53.51% (1249/2334)	52.56% (933/1775)	0.546
High-quality blastocyst (%)	47.73% (1114/2334)	42.31% (751/1775)	<0.001
Biochemical pregnancy (%)	48.31% (86/178)	68.97% (100/145)	<0.001
Clinical pregnancy (%)	43.26% (77/178)	66.21% (96/145)	<0.001
Abortion (%)	10.39% (8/77)	13.54% (13/96)	0.528
Live birth (%)	38.76% (69/178)	57.24% (83/145)	<0.001

Rate of fertilization: the number of fertilized oocytes/the number of matured oocytes.

Rate of cleavage: the number of cleaved embryos/the number of fertilized oocytes.

Rate of high-quality embryo: the number of high-quality embryos/the number of cleaved embryos.

Rate of blastocyst: the number of blastocysts/the number of cleavage embryos.

Rate of high-quality blastocyst: the number of high-quality blastocysts/the number of cleavage embryos.

Rate of biochemical pregnancy: the number of biochemical pregnancies/the number of TET cycles.

Rate of clinical pregnancy: the number of clinical pregnancies/the number of TET cycles.

Rate of abortion: the number of abortions/the number of clinical pregnancies.

Rate of live birth: the number of deliveries with live births/the number of TET cycles.

The euploidy rate of Strategy A was significantly higher than that of Strategy B (35.80% vs 25.28%; *P*<0.001). While the aneuploidy rate (63.35% vs 68.40%; *P*=0.016), mosaic rate (10.45% vs 22.95%; *P*<0.001) and no-results rate (0.93% vs 5.65%; *P*<0.001) were significantly lower than those of Strategy B (**Table 3**).

**Table 3:** NGS results.

	Strategy A	Strategy B	*P*-value
PGT-TET cycles (n)	178	145	–
No. of blastocysts biopsied (n)	1187	902	–
Euploid blastocyst (%)	35.80% (425/1187)	25.28% (228/902)	<0.001
Aneuploid blastocyst (%)	63.35% (752/1187)	68.40% (617/902)	0.016
Mosaic blastocyst (%)	10.45% (124/1187)	22.95% (207/902)	<0.001
No-results blastocyst (%)	0.93% (11/1187)	5.65% (51/902)	<0.001

Rate of euploid blastocyst: the number of euploid blastocysts/the number of blastocysts biopsied.

Rate of aneuploid blastocyst: the number of aneuploid blastocysts/the number of blastocysts biopsied.

Rate of mosaic blastocyst: the number of mosaic blastocysts/the number of blastocysts biopsied.

Rate of no-results blastocyst: the number of no-results blastocysts/the number of blastocysts biopsied.

In terms of clinical pregnancy outcomes, Strategy A’s biochemical pregnancy rate (48.31% vs 68.97%), clinical pregnancy rate (43.26% vs 66.21%), and live birth rate (38.76% vs 57.24%) were significantly lower than Strategy B (*P*<0.001). There was no difference in abortion rate between the two groups (10.39% vs 13.54%; *P*=0.528) (**Table 2**). Infants born with the two biopsy strategies were similar in Apgar score, height, and weight. The average length and weight of newborns in Strategy A were 50.03cm and 3346g, respectively, 50.04cm and 3290g in Strategy B (**Table 4**). Among them, Strategy A had 10 premature babies, including a pair of monozygotic twin daughters. Strategy B had 7 premature babies, including a pair of monozygotic twin daughters and sons. Strategy A had a baby girl born with thumb syndactyly, and Strategy B had no babies with congenital disabilities. The follow-up survey showed that these children were in good health.

**Table 4:** Health of newborns.

	Strategy A	Strategy B	t value	*P*-value
No. of births (n)	70	85	–	–
Apgar score	10.00 ± 0.00	9.95 ± 0.27	1.505	0.134
Weight (g)	3346 ± 529.20	3290 ± 612.00	0.607	0.545
Height (cm)	50.03 ± 2.37	50.04 ± 2.32	0.037	0.971

## 4 Discussion

In the number of cycles we counted, 35 elderly females (≥35 years old) from Strategy A, accounting for 19.67% of the total number of cycles, while 32 elderly females from Strategy B, accounting for 22.07% of the total number of cycles. Advanced age may affect oocyte quality, embryo development potential, ovarian function, hormone level, embryo implantation, and pregnancy (17, 18), which may be why Strategy B had a higher abortion rate than Strategy A, but there was no statistically significant difference.

In terms of embryo development in both groups, it is noteworthy that the rate of high-quality embryos of Strategy A was significantly lower than that of Strategy B, but the rate of high-quality blastocyst was higher. This is very interesting and worth thinking about. We suspected that this might be because Strategy A perforated the ZP on day 3, which was more conducive to embryo hatching. Some hatching blastocysts may actually didn’t fully expand to a morphologic grade of 5 or greater with AA, AB, BA, or BB on day 5 or 6, but the ZP was opened, and the embryos were “squeezed” out, thus increasing the so-called “high-quality hatching blastocyst rate” in Strategy A. Study demonstrated that higher-quality blastocysts could achieve better implantation and live birth rates (8). This may explain why Strategy A had a higher-quality blastocyst rate but unsatisfactory pregnancy outcomes.

As for the biochemical pregnancy rate, clinical pregnancy rate, and live birth rate of Strategy A were far lower than Strategy B, several reasons may explain this phenomenon. Embryonic genome activation (EGA) mainly occurs at the stage of division from 4 to 8 cells (19). Both Dobson and Vassena’s teams demonstrated that the major wave of EGA in human occurs on the third day regardless of the number of cells (20, 21). On the third day, the ZP perforation caused trauma to the embryo during the cleavage stage, and the frequent manipulation of the embryo made the culture environment unstable, which may adversely affect the EGA and be detrimental to the growth of embryos (22). Furthermore, although drilling of the ZP with laser pulses on day 3 could promote early blastocyst hatching, the phenomenon of complete expansion of the blastocyst cavity and thinning of the ZP would not occur during the development of the blastocyst. Additionally, the number of TE cells would also be less than that of non-intervened blastocysts, which could only reach 60-80 cells in total, while non-assisted hatching blastocysts could reach 60-100 cells (23). Sufficient cell numbers could alleviate the negative impact of further reduction of cell numbers caused by biopsy on the results of embryo transfer. Another important reason may have to do with the ICM. Some studies have shown that the natural incubation site of human embryos is near the ICM, so that the embryos are more accessible to implant (24). Due to the randomness of the placement that zona breached by laser, the auxiliary incubation site may be far away from the ICM, thus reducing the chance of embryo implantation after TET. Moreover, if the ICM hatched out, in order to avoid hurting the ICM during the biopsy, a double zona drilling method for ICM incarceration may be used (25). Repeated laser stimulation would inevitably cause adverse effects on the embryo, thereby reducing the embryo quality and affecting the development potential of the blastocyst.

In contrast, Strategy B didn’t damage the embryos during the cleavage stage, which could effectively avoid the potential danger of warming by laser and exposure to a suboptimal environment for a long time. In addition, it was safer that ZP remained intact, which could prevent premature hatching of embryos when the number of cells was small, thus ensuring the normal development of embryos.

The NGS data showed that Strategy A had a higher euploidy rate, while the rate of aneuploidy, mosaic and non-result were lower. Why the NGS analysis results of Strategy A is better? We suspect that the possible reason is that the biopsy subject of Strategy B is contracted TE, which increases the operation difficulty of biopsy, thereby resulting in that the number of TE cells biopsied by Strategy B is generally smaller than that by Strategy A. Therefore, the no-results rate of Strategy B is higher. Contrary to Shun Xiong et al. (26), our results showed that Strategy A had a lower rate of mosaic blastocyst. Mosaic embryos with mosaicism <50% transplantation could lead to a healthy pregnancy, it may be related to reduced implantation rate, increased miscarriage rate, and increased risk of fetal 229 abnormalities (27, 28). There was a significant difference in the rate of mosaic embryo transfer in this study. Strategy A transferred 2 mosaic embryos, accounting for 1.11% of the cycles, while strategy B transferred 10 mosaic embryos, accounting for 6.45% of the cycles. However, the clinical pregnancy outcome of Strategy B was still more ideal than that of Strategy A, consistent with previous studies (29, 30). It demonstrated that Strategy B may be less harmful to the embryos. The study is reporting an experience of a single center, which may be limited in some aspects. More PGT cycles are needed for further exploration.

In a word, the embryos biopsied by Strategy B were more likely to implant and maintain the pregnancy, and the rate of biochemical pregnancy, clinical pregnancy, live birth were much higher than those of Strategy A (about 20%), showing better clinical outcomes. Therefore, based on the above results, Strategy B is an optimal treatment regime for PGT.

## Data Availability Statement

The original contributions presented in the study are included in the article/**Supplementary Material**. Further inquiries can be directed to the corresponding authors.

## Ethics Statement

The biological sample study was approved by the Medical Ethics Committee of Anhui Medical University (Ethics approval number: 2017002). The patients/participants provided their written informed consent to participate in this study.

## Author Contributions

ZZ designed and supervised the study. HY and DY analyzed the data and wrote the main manuscript. HZ and YC performed part of the experiments and revised the manuscript. QZ, KW, and CZ prepared all the figures and videos. BC, WZ, DD, WZ, and DJ performed part of the experiments. YH and DC provided the NGS reports. All authors read and approved the final manuscript.

## Funding

This research was supported by National Natural Science Foundation of China (No.82071724, 32000642 and 82001631).

## Conflict of Interest

The authors declare that the research was conducted in the absence of any commercial or financial relationships that could be construed as a potential conflict of interest.

## Publisher’s Note

All claims expressed in this article are solely those of the authors and do not necessarily represent those of their affiliated organizations, or those of the publisher, the editors and the reviewers. Any product that may be evaluated in this article, or claim that may be made by its manufacturer, is not guaranteed or endorsed by the publisher.
